# The potential of historical ecology to aid understanding of human–ocean interactions throughout the Anthropocene

**DOI:** 10.1111/jfb.15000

**Published:** 2022-02-09

**Authors:** Ruth H. Thurstan

**Affiliations:** ^1^ Centre for Ecology and Conservation University of Exeter Cornwall UK

**Keywords:** environmental history, fisheries, historical ecology, shifting baselines, social‐ecological systems

## Abstract

Marine historical ecology emerged in the scholarly literature with the aim of understanding long‐term dynamics in marine ecosystems and the outcomes of past human–ocean interactions. The use of historical sources, which differ in temporal scale and resolution to most scientific monitoring data, present both opportunities and challenges for informing our understanding of past marine ecosystems and the ways in which human communities made use of them. With an emphasis upon marine social‐ecological changes over the past 200 years, I present an overview of the relevant historical ecology literature and summarise how this approach generates a richer understanding of human–ocean interactions and the legacies associated with human‐induced ecosystem change. Marine historical ecology methodologies continue to be developed, whereas expanded inter‐ and multidisciplinary collaborations provide exciting avenues for future discoveries. Beyond scholarship, historical ecology presents opportunities to foster a more sustainable relationship with oceans going forward: by challenging ingrained perceptions of what is “normal” within marine ecosystems, reconnecting human communities to the oceans and providing cautionary lessons and exemplars of sustainable human–ocean interactions from the past. To leverage these opportunities, scholars must work alongside practitioners, managers and policy makers to foster mutual understanding, explore new opportunities to communicate historical findings and address the challenges of integrating historical data into modern‐day frameworks.

## 1 INTRODUCTION

The importance of oceans for current and future human health and well‐being is of increasing focus for international policy, with significant developments planned over the coming decade to improve ocean health and develop sustainable ocean economies (European Commission, 2017; Food and Agriculture Organisation, 2017; United Nations Environment Programme, 2012; United Nations General Assembly, 2017). This enhanced policy focus is because of increasing recognition of the reliance of many nations and coastal communities upon ocean resources, and the projected future growth in demand for living marine resources (Costello *et al*., 2020). Fisheries and aquaculture currently provide 20% or more of animal protein for 3.3 billion people, supply vital micronutrients and deliver millions of diverse livelihood opportunities (Food and Agriculture Organisation, 2020; Hicks *et al*., 2019). Some of the habitats that support the provision of fish protein are also capable of delivering significant levels of carbon sequestration and a host of wider ecosystem services of benefit to human health and well‐being (Barbier, 2017; Krause‐Jensen & Duarte, 2016; Macreadie *et al*., 2014).

Although such policy initiatives present opportunities for new research and practice, they have been introduced against a backdrop of accelerating marine habitat degradation and biodiversity loss (Sala & Knowlton, 2006). It is increasingly accepted that humans have had significant and long‐term impacts upon marine ecosystem structure and functioning (Díaz *et al*., 2019). What remains less clear is the magnitude of ecological changes to have occurred as a result of human impacts, to what extent we can reverse such changes, the trade‐offs required to do so and the benefits that would result (McClenachan *et al*., 2012; Thurstan *et al*., 2015; zu Ermgassen *et al*., 2020). Our inability to adequately answer these questions shows a need for interdisciplinary approaches and tools to incorporate historical perspectives and lessons into planning and policy initiatives (Alleway *et al*., 2016; Caswell *et al*., 2020; Engelhard *et al*., 2016).

Part of the disconnect between our long history of transforming marine ecosystems, and the timely recognition of these impacts, is the difference between the lengths of time over which marine ecosystems have been exploited and the period spanned by scientific monitoring. By the time the sciences turned their attention to marine ecosystems (Hubbard, 2017; Smith, 1994), humanity had already established a long history of changing them. In the Atlantic Ocean, fleets of fishing vessels from Europe exploited the Grand Banks of the Western Atlantic from the 16th century onwards (Holm *et al*., 2019; Nicholls *et al*., 2021), whereas evidence of a North European transition from freshwater to marine fish consumption occurred centuries earlier (Barrett *et al*., 2004). By 1800, salted, cured and live fish products had been traded for centuries, with species such as herring (*Clupea harengus*) and cod (*Gadus morhua*) supporting a thriving international fish trade (Barrett *et al*., 2011; Orton *et al*., 2014; Poulsen, 2008). Many coastal economies were also well developed, with evidence of localised impacts emerging in the pre‐industrial period (Jones *et al*., 2016). As the industrial period commenced, land use and coastal development enhanced silt and pollutant loads in many estuarine and inshore systems (Kemp *et al*., 2015; Lotze *et al*., 2006). The 19th‐century emergence of rail transport networks and facilities to store ice opened up trading opportunities for perishable fish goods (Robinson, 1996). At the same time, growing urban populations created a rising demand for cheap protein, facilitating the expansion and further innovation of technologies that could supply large quantities of fish, including mobile bottom fishing gears (Robinson, 1996; Thurstan *et al*., 2014). Throughout the 20th century, demand for fish continued to grow, and many fishing operations became more mechanised and intensive, with some vessels capable of spending months at sea catching and processing fish for national and international markets (Pauly *et al*., 2002; Swartz *et al*., 2010).

In short, humanity has a long history of degrading and altering marine ecosystems (Ellis *et al*., 2010; Scheffer *et al*., 2001; Tucker *et al*., 2018), yet our ability to observe and monitor these changes commonly lagged behind (Cushing, 1988; Smith, 1994). In the case of fisheries, technological developments take place incrementally and with variable impacts across target species, making such changes extraordinarily difficult to quantify without significant and sustained monitoring effort (Engelhard, 2008; Engelhard, 2016; Palomares & Pauly, 2019). These developments also took place as fisheries intensified and expanded their reach (Watson *et al*., 2013). Together, these confused the signals from readily collated forms of monitoring data: fish landings and landings per unit of fishing effort (Swartz *et al*., 2010; Thurstan *et al*., 2014; Watson *et al*., 2013). As a result, we tend to approach current challenges in marine ecosystem recovery largely ignorant of the long history of human depredations upon them (Bolster, 2006; Jackson *et al*., 2001). Indeed, basic knowledge such as what marine ecosystems used to look like, natural ranges of variability and how ecosystems functioned before significant human‐driven changes is often missing (Klein & Thurstan, 2016; McClenachan *et al*., 2012; zu Ermgassen *et al*., 2012).

Together with our inability to directly observe ecological changes below the waters’ surface until recent decades, the above factors have resulted in a collective historical unawareness of past marine ecological changes across generations of resource users, scientists and managers. This results in intergenerational shifts in peoples’ expectations of how “natural” ecosystems should look and function, popularly termed the shifting baseline syndrome (Pauly, 1995). Although the shifting baseline syndrome can occur in any system that undergoes changes across multiple human generations (Hanazaki *et al*., 2013; Papworth *et al*., 2009; Sáenz‐Arroyo *et al*., 2005), it is particularly prevalent in marine ecosystems as the history of ecosystem change is largely unknown (Pauly 1995; Dayton *et al*., 1998; Pinnegar & Engelhard, 2008). To counter this, attempts are increasingly being made to understand this history using palaeoecological, archaeological and historical evidence (Braje *et al*., 2017; Lotze *et al*., 2006; Pandolfi *et al*., 2003; Rick & Lockwood, 2013). Through such studies, we are beginning to discover the timing, direction, magnitude and drivers of change within marine systems across past decades, centuries and millennia, and are becoming aware of the profound impacts our land‐ and ocean‐based activities have had upon the structure and functioning of marine ecosystems (Jackson *et al*., 2001; Pinnegar & Engelhard, 2008).

In the following sections, I present an overview of the marine historical ecology literature to highlight the ways in which historical sources have been applied to generate a deeper understanding of past marine ecosystem dynamics and human–ocean interactions. I suggest ways in which historical perspectives can help to foster a more sustainable relationship with our oceans going forward, and I argue for further development and integration needs in marine historical ecology. Given the emphasis upon the Anthropocene in this special issue, this paper restricts its focus to studies of social‐ecological changes covering the past 200 years. For archaeological and material collections, I refer the reader to a comprehensive review by Barrett (2019, this journal) and references therein. For an introduction to the field of environmental history, see Schwerdtner‐Máñez and Poulsen (2016) and Christensen and Tull (2014). My own disciplinary limitations restrict this review to research that interrogates documentary and oral evidence. Limiting the scope of the paper to the most recent two centuries precludes a description of the important history and outcomes of pre‐industrial human–ocean interactions (*e.g*., Holm *et al*., 2021), but this recent period remains highly significant as a time of accelerating ocean impacts that resulted in profound ecological changes, the legacies of which continue today.

## 2 THE EMERGENCE OF MARINE HISTORICAL ECOLOGY

Marine historical ecology emerged in the late 20th century in response to concerns that scientific data collection occurred over too short a time period to comprehend the scale of human‐induced changes to marine ecosystems (Dayton *et al*., 1998; Jackson *et al*., 2001). Overlapping with the field of marine environmental history and sitting at the intersection of a number of disciplines, including archaeology, history, anthropology and palaeoecology, marine historical ecology involves scholars and practitioners from the humanities, the natural and the social sciences. Consequently, research conducted under the umbrella term of “marine historical ecology” is highly diverse in terms of the questions asked, the time scale and spatial scale of focus, the sources chosen for interrogation and the analytical techniques used (Kittinger *et al*., 2015; Schwerdtner‐Máñez & Poulsen, 2016). Historical ecology investigations may cover time scales from a few years in the past, to centuries, or even millennia. For example, palaeoecological investigations have significantly expanded our understanding of the distribution, community composition, functioning and dynamics of change in marine ecosystems before human contact, as well as the timing of human‐induced ecosystem change (Froyd & Willis, 2008; Yasuhara *et al*., 2012). Archaeological investigations have shown the longevity of human marine resource use, the pre‐industrial impacts of marine exploitation and the levels of dependence of past human societies upon marine ecosystems (Bailey, 1975; Barrett, 2019; Barrett *et al*., 2004; Orton *et al*., 2014; Rick *et al*., 2016). Historical, anthropological and ethnographic sources have provided detailed understanding of the type, scale and consequences of human activities for marine ecosystems over the past centuries up until the present day (Armstrong *et al*., 2017; Jackson *et al*., 2001; Kittinger *et al*., 2015; Pandolfi *et al*., 2003; Roberts, 2007; Tushingham *et al*., 2020).

With the potential to generate a clearer understanding of past ecosystem dynamics, the impacts of human interactions and wider system feedback, marine historical ecology presents an opportunity to understand not only the *how* and *when* (*how* have our ecosystems changed, and *when* did these changes occur?) but also the *why* and *what* (*why* did these changes occur, and *what* social‐ecological feedbacks resulted?). In so doing, this approach can provide new perspectives not only on the health of marine ecosystems today, but also on the ways in which human communities have behaved, valued and interacted with marine ecosystems through time, the complex feedbacks that have resulted, and how the legacy effects of past human–ocean interactions continue to resonate today (MacDiarmid *et al*., 2016; Schwerdtner‐Máñez *et al*., 2014).

## 3 UNDERSTANDING ECOSYSTEM CHANGE AND HUMAN–OCEAN INTERACTIONS

**FIGURE 1:**
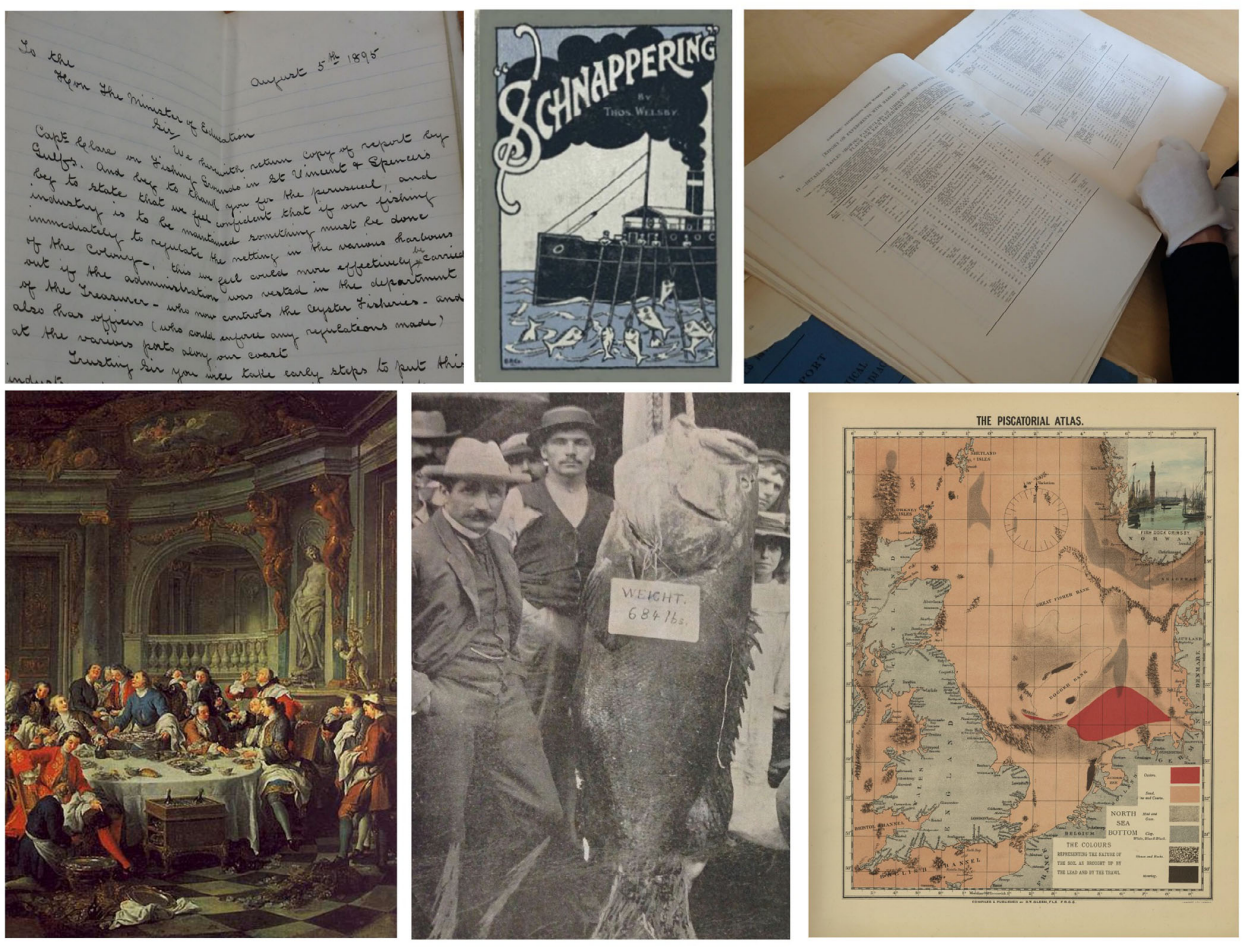
Examples of written materials and images used to understand historical marine social‐ecological systems. Clockwise from top left: Letters and other forms of correspondence provide insights into contemporary concerns regarding exploitative practices and their impacts upon marine populations (picture: RH Thurstan); popular media can inform when activities such as recreational fishing intensified, locations fished and the catches that occurred (Welsby, 1905); government documents provide data on historical patterns of exploitation (picture: ES Klein); nautical charts provide an indication of the location and extent of certain marine habitats (Olsen, 1883); pictures and newspapers provide insights into species occurrence and size, as well as the frequency with which they were observed or caught (“A RECORD FISH,” 7 October 1899, The Queenslander, Brisbane, Queensland, p 714, National Library of Australia); artwork can highlight the use of marine species and their cultural significance (Jean François de Troy, Oyster Lunch, 1735. Public domain, *via* Wikimedia Commons)

**FIGURE 2:**
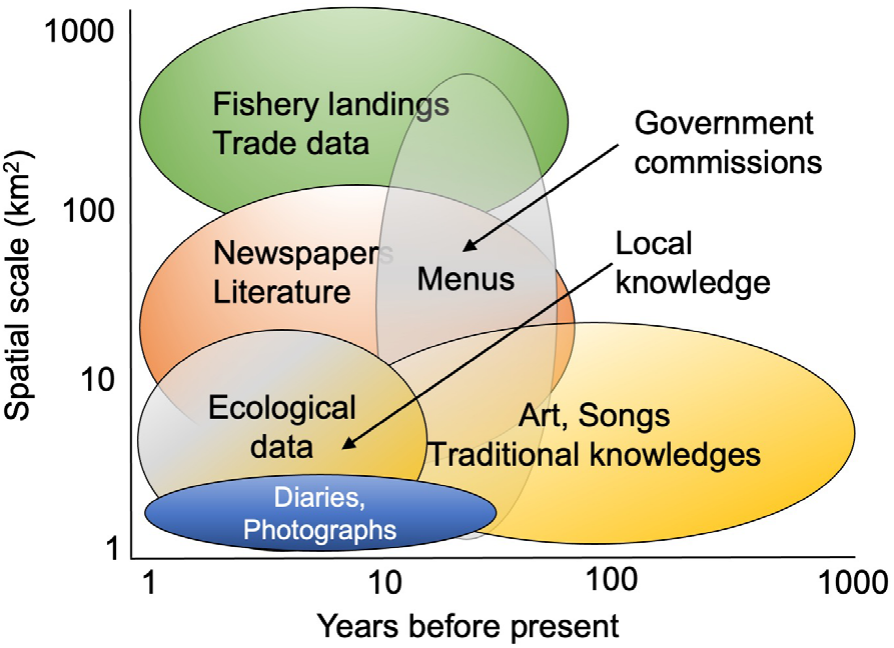
The potential temporal and spatial scales that can be covered by selected forms of historical data used in marine historical ecology research

Historical sources used to understand system change include written materials, imagery and oral accounts, sourced from public, government and institutional libraries, museums, national and regional archives, individual record keepers and private collections. Examples of written materials include landings and trade records, fisheries time series, popular literature and media such as naturalist and traveller accounts, newspaper articles and household records and menus (Fortibuoni *et al*., 2010; Poulsen, 2010; Sáenz‐Arroyo *et al*., 2006; Thurstan *et al*., 2010; Van Houtan *et al*. 2013). Imagery includes artwork, photographs and nautical charts (McClenachan, 2009; McClenachan *et al*., 2017; Mojetta *et al*., 2018; Thurstan *et al*., 2013) (Figure 1). Oral accounts include traditional, Indigenous and local ecological forms of knowledge (Sáenz‐Arroyo *et al*., 2005; Shackeroff *et al*., 2011; Buckley *et al*., 2017; Thurstan *et al*., 2018; see Drew, 2005 for definitions). The temporal scale of these historical sources can span years, decades or even centuries depending upon the preservation of records and the consistency of reporting, with some Indigenous knowledges, songs and other expressions of cultural memory being found to span thousands of years (Nunn & Reid, 2016) (Figure 2).

The scale, resolution and context within which historical sources were produced impact the extent to which findings can be generalised across temporal and spatial scales (Figure 2), as well as the type of questions that can be asked of the available evidence. When using past sources, historical ecologists adopt long established methods of source handling and criticism from historical disciplines (*e.g*., Poulsen, 2016 and examples therein). The human‐centred nature of historical documentation means that the social and cultural contexts of historical sources and past periods need to be understood, including how and why the evidence was recorded in the first place, to interpret signals of ecological change (Poulsen, 2010; Schwerdtner Máñez and Poulsen, 2016). Moreover, historical documents rarely provide direct observations of marine ecosystems and changes through time. Instead, most provide glimpses of past ecosystems filtered through a lens of contemporary concerns, societal norms and cultural interests, providing rather noisy and indirect indicators of change, such as occurrence data, landings or catch rates (Lotze & McClenachan, 2013).

Historical records also tend to emphasise species that were readily observable or of high cultural or commercial interest, whereas marine habitats were preferentially mapped according to their perceived use or danger to navigators (Christensen, 2014; McClenachan *et al*., 2017; Thurstan *et al*., 2020). Historical sources may also be subject to changes in reporting that could impact the comparability of time series data. For example, species (not) included in commercial landings data may change due to regulatory requirements or shifts in market demand (Alleway *et al*., 2014), whereas the consistency of sightings or content of fishing reports may change as the newsworthiness of particular events alter over time (Thurstan *et al*., 2018). It is therefore necessary to understand the wider social, political and cultural systems that existed during the period of interest and how these resulted in particular events being recorded (or not), and to interpret the available historical evidence through these wider lenses.

Many different forms of data have been extracted from historical materials to contribute to our understanding of marine ecosystem dynamics, including past species occurrence and distribution, population dynamics and changes to ecological functioning. Historical sources also provide insights into the drivers and human perceptions of change through time, including the ways in which human communities drove, responded and adapted to change, how we perceived and valued marine ecosystems and how cultural and social norms affected our interactions with marine ecosystems through time. The following sections provide an overview of studies that have used historical sources to generate such insights.

### 3.1 Changes in species occurrence and distribution

Marine historical ecology continues to challenge widely held assumptions across the marine sciences regarding the occurrence and distribution of marine habitats and species. Studies of biogenic shellfish habitats often point to the presence of once‐vast and complex habitats, now much reduced or even rendered locally extinct (Kirby, 2004; Thurstan *et al*., 2014; Alleway & Connell, 2015, Bennema *et al*., 2020; Thurstan *et al*., 2020; Box 1). Historical evidence also points to significant changes in the occurrence and distribution of non‐biogenic marine habitats, forage fish and various species of megafauna resulting from exploitation, habitat destruction, pollution or climate‐induced changes (*e.g*., Ames, 2004; Dulvy *et al*., 2016; Green *et al*., 2021; Hall *et al*., 2012; Kittinger *et al*., 2013; McClenachan *et al*., 2006; McClenachan & Cooper, 2008).

BOX 1 **Historical distribution of habitats and species**

Native oysters (*Ostrea edulis*) are one of the most highly threatened marine habitats in Europe, but information to support restoration action, such as where oyster habitats used to exist and historical levels of abundance, is lacking (Preston *et al*., 2020). The social and economic significance of native oysters over the centuries has, however, resulted in a wealth of historical sources that can help fill these knowledge gaps. *Sources* include witness statements recorded during parliamentary inquiries, popular publications, maritime charts, scientific investigations and annual fisheries statistics, among others (Figure 1).

*Information extracted* may include the location or name given to exploited oyster habitat, spatial delineations of known habitat and estimates of the numbers of oysters extracted in a given period, with such information rarely consistent across locations. Occurrence data may take the form of brief mentions of the presence of oysters, “There are several natural oyster beds in Broadhaven and Blacksod bays” (Irish Fisheries Report, 1836 p 82), to more detailed accounts of the location of oyster habitat, “…There is a noble (oyster) bank or bed betwixt Laxey Bay and Maughold Head extending above two miles in length and near two in depth…about a mile and a half from the shore” (Townley, 1791 p 303). Sources also offer insights into when specific habitats began to be exploited, “In the autumn of last year a large bed of oysters was discovered between the Ridge and the Brixey Sands, at the mouth of the Crouch, Essex” (Buckland, 1875 p 22), and instances of overexploitation, “There was an immense haul of oysters at the Dudgeon Light, that bed was found 40 years ago, and worked off within three or four years, and there have not been any considerable number of oysters there since that time” (Select Committee, 1876 p 124).

*Critical considerations* when interrogating sources to determine past presence and distribution of species or habitats include: are certain species or locations likely to be under‐ or overrepresented in the sources chosen for interrogation? Who is providing this information, and are they likely to have a vested interest in misrepresenting the scale or location of a species? Are there multiple, independent primary sources verifying occurrence? Are these descriptions first‐hand, and are they referring to contemporary or past occurrence? For all these questions, consideration of the biases that may exist allows us to assign relative levels of confidence to the accuracy and precision (and the likelihood of missing data) of historical distribution records.

Even brief snippets from history can indicate past presence. In some cases, the historical record may concede nothing more than the mention of a species or habitat type in a region. In other instances, historical narratives provide rich detail on the extent and form of a marine habitat, or the distribution of a species (Box 1). At times, documented locations cannot be judged with any great precision, although some habitats, such as shallow coral reefs that posed a danger to navigators, were described in high resolution (McClenachan *et al*., 2017). Much harder to ascertain from historical records is the presence or absence of species that were *not* of cultural interest, or species that were rare or in decline at the time of writing. For marine species that were not commercially or culturally important, their appearance within the historical record may underestimate past distribution (Thurstan, Pandolfi *et al*. 2016; Christensen, 2014). Similarly, species that were in decline or rare by the time written records started to be kept may have been overlooked in contemporary writings. In such cases, studies that combine archaeological with historical evidence can show if historical timelines present an already‐shifted baseline (Barrett, 2019).

### 3.2 Population dynamics and relative abundance

Proxies of population dynamics, such as changes in relative abundance, can be inferred from some historical sources (Box 2). Records may take the form of landings, catches or catch rates, or perceived changes in abundance recorded by contemporary observers, and be expressed quantitatively or qualitatively (Palomares *et al*., 2006; Thurstan *et al*., 2014). In most cases, trends in absolute abundance are difficult to obtain (although see Poulsen *et al*., 2007). More commonly, catch rates and landings are extracted (Thurstan, Campbell *et al*. 2016; Poulsen, 2010; Ulman *et al*., 2020) or relative abundance estimated from qualitative or semi‐quantitative evidence (Fortibuoni *et al*., 2010; Palomares *et al*., 2006; Pandolfi *et al*., 2003) and used to infer changes through time. The prevalence of non‐linear dynamics in fish populations before and after exploitation, and the feedbacks between exploitation and non‐linear dynamics for population resilience, is also beginning to be explored (*e.g*., Klein *et al*., 2016).

BOX 2 **Past fisheries and fishing trends**

Patterns of exploitation can be driven by environmental shifts, changes in species abundance, and economic, technological and/or cultural factors. Understanding the early years of a fishery's exploitation and its impacts upon habitats and populations can be informative for stock assessment and management, but early monitoring data are rare. *Sources* on past fisheries include government inquiries, newspaper and magazines, interviews, maritime charts and commercial records.

*Information extracted* may include locations fished, quantity of fish caught, inter‐sectoral conflict, the introduction of new technologies and societal transitions. For example, records of recreational fishing trips exist in newspapers, which illuminate past catch rates that can be compared over time, “At a little before 7 we steamed up to the Boat Rock, down went about twenty‐four lines; in two minutes the cry rose ‘schnapper’ (…). For four hours and a quarter the sport was sustained (…). We were found to have lessened that particular tribe of schnapper by about 735 individuals” (The Queenslander 24 May 1879, p 655). These articles and interviews with fishers also highlighted the type and timing of social transitions that influenced the interpretation of time series trends, “In the 1970s and ‘80s people would fish for the 30 bag limit because they could get away with selling the fish, now most want to preserve stocks” (Interview with charter fisher in 2015, Brisbane, Australia; Thurstan *et al*., 2018). The impacts of earlier technological change can also be extracted. For example, the 19th‐century expansion of demersal trawling resulted in national‐level government inquiries. Occurring before the collation of national fisheries statistics, these inquiries provide rich narrative detail of the ecological degradation that was reported as trawlers targeted new grounds, “I believe there is not a portion of the ground but what the trawl destroys (…) (I have) brought up coral about 2.5 feet in circumference, lumps of soft coral, and I am prepared to say that whatever is in the way of the beam trawler will not escape” (G Cormack, line fisher and ex‐trawler, Royal Commission 1885 p 11). They also showed changing patterns in exploitation, “When I first went to sea the nearest fishing grounds to the mouth of the river Humber were distant from between 30 and 40 miles (…) the nearest fishing grounds of any note are now distant from the Humber 170 miles” (JL Potter, Smack Owner, Royal Commission 1885 p 254) and emphasised the differing perceptions across sectors that led to conflict, “…as the crow follows the plough for the worm, so the stirring of the ground brought the fish, and made our fishing ground really prolific, a beautiful provision of nature” (J Bartlett, Fishery Board Chair, Royal Commission 1885, p 324).

*Critical considerations* when interrogating past fishery data include: Who reported these observations and did they have reason to exaggerate or understate their testimony? Do independent data exist to determine levels of source or individual bias? Who had their testimony recorded and under what circumstances, and what perspectives may be missing from the documentary record? That is, were successful recreational fishing activities preferentially recorded, and were fishery inquiry witnesses pressured to present a particular version of events? Answering these questions can help determine whether observed trends primarily reflect ecological, social or geopolitical changes.

Historical context is particularly important when considering whether the above trends reflect real changes in species abundance. Additional data such as evidence for changes in the market value of a species, the timing of technological innovations or regulations or spatial shifts in fishing effort can help to inform whether a trend potentially masks or exaggerates real change (Ames, 2004; Buckley *et al*., 2017; Fortibuoni *et al*., 2017; Jacobsen, 2014). Although these are common considerations for quantitative data such as landings, catches and catch rates, such factors also influence qualitative evidence such as historical descriptions of abundance. For example, enhanced accessibility due to technological innovation is likely to influence resource users’ perceptions of the relative abundance of a target species, as will intergenerational shifts in the perception of what a natural ecosystem can produce (Sáenz‐Arroyo *et al*., 2005). The meanings and uses of words and phrases can also vary over time and space, or be interpreted differently by individual researchers. None of these issues are insurmountable and are sometimes measurable (*e.g*., Al‐Abdulrazzak *et al*., 2012; Sáenz‐Arroyo *et al*., 2005), but they point to the necessity of considering the historical context of data, as well as the disciplinary and cultural biases of the person(s) interrogating the data.

### 3.3 Marine communities and ecological functioning

Historical records have highlighted marine community change and the resulting impacts upon ecological functions, feedback and ecosystem services (Lotze, Coll, & Dunne, 2011). For example, research into historical abundances of anadromous forage fish in Maine showed an almost complete loss of historical productivity within contiguous watersheds (Hall *et al*., 2012). These impacts cascaded to predator species within the system and greatly reduced overall ecosystem productivity, demonstrating the need to account for changes to wider ecosystem functioning and specifically, predator–prey relationships when exploring restoration potential (Hall *et al*., 2012). Research into benthic communities has also shown significant community shifts. For example, in the North Sea, bivalve frequency of occurrence was found to decline throughout the 20th century compared to the frequency of scavenger and predator species, which markedly increased over the same time period (Rumohr & Kujawski, 2000). Research has also identified the significance of past ecosystem services provided by historical populations, such as the locations where bivalve filtration historically had measurable impacts on water quality, and hence where restoration to historical abundances would likely lead to estuary‐wide improvements (zu Ermgassen *et al*., 2013).

### 3.4 Drivers of ecosystem change

Exploring change across broad temporal scales can help to disentangle the major drivers of change, either quantitatively or qualitatively. Studies focused on multi‐decadal shifts in species distribution or degradation have assigned observed changes to habitat alteration, climate change and/or fishing pressure (Dulvy *et al*., 2016; Engelhard *et al*., 2014; Hall *et al*., 2012). Changes in the dominant species of fish landed have been ascribed to environmental, ecological and societal changes (Alleway *et al*., 2014; Van Houtan *et al*., 2013; Gaumiga *et al*., 2007). Changing prices can indicate increased rarity or accessibility of a resource, but may alternatively point to rising supply costs or shifts in consumer preference (Holm *et al*., 2019). Legislative introductions can indicate societal concerns for the persistence of a population, or government interest in regulating or taxing a resource (Kirby, 2004).

### 3.5 Patterns of exploitation

Historical sources have been used to assess changing patterns of exploitation through time by quantifying the rate of adoption of new technologies, increases in the fishing power of vessels and changes in the spatial activity of fleets (Anticamara *et al*., 2011; Swartz *et al*., 2010; Thurstan *et al*., 2010). These patterns provide insights into when certain locations, species and habitats became accessible to exploitation and when exploitation of a population intensified (Box 2). Often, the quantification of these changes and their impacts upon populations is complex (Engelhard, 2016; Palomares & Pauly, 2019), but historical insights can aid our understanding of temporal and spatial patterns of exploitation and hence the interpretation of time series trends, as well as the likely ecological outcomes (Buckley *et al*., 2017; Jacobsen, 2014). For habitats known to be vulnerable to degradation from mobile fishing gears, knowledge of the timing of technological adoption can help determine whether historical sources were describing an already much‐altered ecosystem.

### 3.6 Social and cultural values

Marine historical ecology and environmental history are informing the ways in which past communities valued the seas and the species therein, how such values have altered with time and the outcomes of these (Mazzoldi *et al*., 2019; Pepin‐Neff, 2014). Information on historical social and cultural values is also highly pertinent to understanding the ways in which marine species and spaces were valued in the past, as these will influence the quantity, quality and narrative forms of reporting across the historical record. For example, many marine species were written about because they were valued as food or as other types of resource provision (*e.g*., fuel, bedding and manure), whereas some species attracted recordings or comment because of their trade significance or association with taxation. Still other historical writings highlight the occurrence of particular species because of their strange forms and inquisitiveness or because of the fear associated with them (Brito *et al*., 2019). Some fishing gears and their ecological impacts were reported on widely because of long‐standing controversy over their use (*e.g*., Sea Fisheries Commission, 1866; Box 2), whereas catches or landings were reported because a species was culturally significant or recreationally prized, or because particular events were deemed newsworthy (*e.g*., Thurstan, Campbell *et al*. 2016; Thurstan *et al*., 2018).

As such, historical sources reveal what past human communities valued enough to write, draw and talk about. Such values are multifaceted and challenging to isolate and quantify, but they cannot be ignored. Nor can changes in social and cultural values through time, or how they vary across communities. Cultural and social values also shift as a result of new legislation, knowledge or technology, or because of wider societal change. Such changes may occur rapidly – within the course of a few years – or across multiple generations. Examples include the shift from “take all” to “catch and release fishing” in Australian culture (Frawley, 2015), the shift from seeing sharks as “monsters” to “charismatic” (Mazzoldi *et al*., 2019) and movement away from an extractive towards a conservation mindset by scuba divers and spear‐fishers (Whatmough *et al*., 2011; Young *et al*., 2014).

Such social and cultural values are important to understand because they impact what was recorded in historical documents. Social and cultural norms and values also provide us with clues as to what might be missing from the historical record: a lack of visibility in historical documentation of certain resource users (*i.e*., women, Indigenous groups) is common and can lead to an underestimation of historical resource use (*e.g*., species upon reef flats are often predominantly gleaned by women and children, Grantham *et al*., 2020). Although still an emerging area of research, exploration of cultural values also helps us to understand the complex contributions of marine ecosystems to human well‐being, how these contributions change through time and how the scale and type of benefits we receive are related to marine ecosystem health (Klain & Chan, 2012).

### 3.7 Human responses to change

Our understanding of past human responses to acute and chronic ecological changes and the feedback between societal and ecosystem elements remains poorly resolved. In‐depth analyses are often required to fully appreciate the complex and entangled social, scientific, political and economic factors that drive marine ecosystem and coastal community transformations. For example, McKenzie (2011) demonstrates how centuries of subsistence fishing by the local communities of Cape Cod, USA, were overwhelmed by a complex combination of changing labour and economic regimes that led to ecological degradation and the subsequent transformation of this coastal community away from fishing (McKenzie, 2011). In addition to better understanding *how* past human communities responded to change, history provides cautionary lessons (Caswell *et al*., 2020). For example, societal memory of the subsistence inshore fishery community of Cape Cod was subsequently erased by popular aesthetic, but historically inaccurate, portrayals of the supposedly “pristine” nature of Cape Cod: a nature that had, in fact, lost its once‐abundant fish populations and the active fishing communities they supported (McKenzie, 2011). Such histories also aid our understanding of the likely nature of future adaptation strategies and expected scale of variation across societal responses (Alexander *et al*., 2017; Perry *et al*., 2011), providing meaningful information to aid future planning under environmental or geopolitical change. For example, Alexander *et al*. (2017) demonstrate how the 1816 eruption of Mount Tambora's in Indonesia, and the subsequent (temporary) environmental impacts upon fish stocks on the east coast of the USA led to societal responses that facilitated permanent shifts in the social‐ecological system of the Gulf of Maine.

## 4 USING HISTORY TO FOSTER SUSTAINABLE HUMAN–OCEAN INTERACTIONS

Historical ecology and environmental history research is illuminating the form and functioning of past marine ecosystems, and the magnitude of social‐ecological changes that have occurred. Nevertheless, the ways in which the historical record can be used to foster sustainable interactions moving forward is often less appreciated. Below, I summarise examples where historical perspectives have been shown, or have the potential, to create impact beyond scholarly knowledge.

### 4.1 Challenging perceptions

To what extent our growing knowledge of past ecological change has succeeded in challenging societal expectations of what constitutes a healthy marine ecosystem (*i.e*., reducing the prevalence of the shifting baseline syndrome) has yet to be quantified. The fact that the shifting baseline concept is increasingly researched and referred to in the popular media suggests it is beginning to influence scientific and public perceptions (Engelhard *et al*., 2016; Soga & Gaston, 2018). Yet, the number of people within marine‐facing industries, who interact with the oceans for recreational or cultural activities, or who work within marine policy and management who are aware of the concept of the shifting baseline syndrome, and how such awareness influences behaviours and decision making, remains unknown.

Regardless of these uncertainties, history continues to challenge scientists’ and practitioners’ perceptions of what population sizes, extent, functions and ranges of variability are “normal” within marine ecosystems, and hence what is possible to aspire to. Some inshore habitats have been so dramatically altered in recent centuries that little to no scientific or cultural memory remains as to their past composition and the benefits that coastal communities historically accrued from them. For example, few people today are aware of the past existence and size of oyster reefs, the significant provisioning resource they provided and just how rapidly oysters went from being a foundation species of many coastal ecosystems to being almost completely eradicated from the systems they once defined (Alleway & Connell, 2015; Thurstan *et al*., 2014). Historical perspectives thus promote valuable debate as to what goals we should aim for when managing, conserving or restoring marine ecosystems (Kittinger *et al*., 2015; McClenachan *et al*., 2015). They also help us articulate the societal and economic trade‐offs that would have to occur to return to certain ecosystem states or population sizes (Tomscha & Gergel, 2016). For example, identifying species for which mobile fishing gear or coastal pollution is incompatible with their restoration. The past does not predict the future, but it can give us an indication of when an ecosystem moves outside of its historical range of variability, or when influences upon ecosystems move from being dominated by natural to human drivers (Engelhard *et al*., 2014; MacKenzie *et al*., 2011; O'Dea *et al*., 2020). As such, history can also contribute to averting undesirable social‐ecological shifts, such as the over‐simplification of ecosystems and resulting reliance by fishing communities on a single species (Howarth *et al*., 2014).

### 4.2 Reconnecting human communities with the oceans

Marine ecosystems are increasingly considered social‐ecological systems, *i.e*., systems composed of human and natural elements that are intrinsically linked and interdependent. An emerging literature explores how enhanced connections with an ecosystem or location can foster a sense of stewardship and pro‐environmental behaviour (Berkes *et al*., 2003; Masterson *et al*., 2017; Walker *et al*., 2004). Historical perspectives potentially have important roles to play in furthering our connections with, and hopefully stewardship of, the seas. Firstly, historical perspectives demonstrate our long and continuing connections and reliance upon coastal and marine ecosystems. Secondly, the rich narratives present in historical documents provide scope for engagement with stakeholders (McAfee *et al*., 2020). Place‐based histories can be particularly powerful in connecting communities with their local marine environment, while species‐focused histories form a powerful narrative when the species is culturally important to a stakeholder group or community (Larson *et al*., 2013; Thurstan *et al*., 2020). Historical perspectives also help disentangle popular representation from reality, including when idealised (and long defunct) representations of an industry or location are adopted with the aim to sway public opinion and decision making (McKenzie, 2018). Thirdly, historical ecology can contribute to communicating the wider benefits of healthy marine ecosystems beyond the provision of food and recreation. An understanding of how marine ecosystems contributed to individual and community health and well‐being in the past has great potential to aid understanding of the provision of marine ecosystem goods and services across wider society, today and into the future (Costanza *et al*., 2014; Tomscha & Gergel, 2016).

### 4.3 Cautionary lessons and exemplars from history

The history of past ecosystems and human interactions can provide lessons and cautionary tales regarding the known outcomes of past impacts and decision‐making (Engelhard *et al*., 2016; Lotze *et al*., 2014). Conversely, historical perspectives can also be used to show good practices from the past, such as when communities sustainably exploited and managed local marine ecosystems over long periods of time and how this was achieved (Caswell *et al*., 2020; McClenachan & Kittinger, 2013), how communities adapted to sudden ecological or societal change (Alexander *et al*., 2017) or the conditions required to implement positive changes for societies and ecosystems (Lotze, Coll, Magera, *et al*., 2011). Although the contemporary significance of decisions or social‐ecological changes made long ago, and their outcomes, may not be immediately obvious – particularly in the face of rapid, human‐induced climate and ecological change – an understanding of social‐ecological feedback and the range of human responses to past challenges can provide useful insights for planning and policy as we navigate emerging environmental and ecological challenges (Caswell *et al*., 2020; MacDiarmid *et al*., 2016; Schwerdtner‐Máñez *et al*., 2014).

## 5 FUTURE CHALLENGES FOR THE STUDY OF PAST OCEANS

In many ways, the study of past oceans is only just beginning. We are increasingly learning from history about the form and function of past ecosystems, but this is often limited to well‐documented species and locations. Understandings of change as experienced by historically marginalised communities or groups, and how marine resource use differed across cultures, locations and time periods, and the ecological outcomes of these, remain lacking. As such, further integration of social science perspectives, alongside collaborative, multi‐method approaches to ensure the equitable inclusion and representation of multiple resource user histories, remains key areas for further development in marine historical ecology research (*e.g*., Shackeroff *et al*., 2011; Tushingham *et al*., 2020). In particular, significant knowledge gaps relate to non‐Western histories. In countries with a history of colonisation, colonial perspectives were preferentially recorded by those in power, whereas Indigenous cultures, their histories and methods of historical preservation were actively destroyed. This presents modern‐day researchers with a far less diverse – and often misrepresentative – understanding of the multiple ways in which past oceans were valued, exploited and managed (McClenachan & Kittinger, 2013; Shackeroff *et al*., 2011). As historical evidence becomes more accessible *via* the digitisation of national and local archives, equitable partnerships and the co‐production of knowledge with traditional owners will greatly enhance our understanding of the diversity of past marine resource use, knowledge and management systems (Reid *et al*., 2021).

Although marine historical ecology approaches have demonstrated conceptual advances through inter‐ and multidisciplinary working, there is room for greater attention to this type of work and what best practices can achieve (McClenachan *et al*., 2015). Further acknowledgement and incorporation of theory and methods from wider disciplines will continue to improve our historical understanding and explanations of change. The greater integration of social, cultural and ecological dynamics within the framing of social‐ecological systems perspectives will aid broader understanding of the complex and non‐linear links and feedback between human and non‐human systems, such as the social and physiological adaptations by whale societies to industrial‐scale whaling activities (Trumble *et al*., 2018; Whitehead *et al*., 2021).

To leverage scholarly and applied opportunities, historical ecologists must work alongside scholars from other disciplines, knowledge holders, practitioners, managers and policy makers to foster mutual understanding, explore new opportunities for data application and address the challenges of integrating historical data into modern‐day frameworks (MacDiarmid *et al*., 2016). This includes the potential for the incorporation of deeper historical perspectives into traditional tools, such as fisheries stock assessments, but there is also much scope for historical perspectives to be incorporated into emerging management and policy, including ecosystem‐based management, blue growth and ecosystem services frameworks (Caswell *et al*., 2020; Engelhard *et al*., 2016; Urlich & Handley, 2020). There is also opportunity to use historical perspectives as an engagement tool to enhance knowledge exchange across stakeholder groups, and *via* initiatives aimed at enhancing well‐being and/or ocean literacy. The diversity of available historical evidence and its potential applications means that real‐world application has progressed on a case‐by‐case basis to date (Engelhard *et al*., 2016). As examples of the successful incorporation of historical perspectives increase and best practices become known, the potential of historical ecology research to support the implementation of policy should become more widely recognised and accepted (Beller *et al*., 2020; Raicevich, 2013).

## 6 CONCLUSIONS

Marine historical ecology research demonstrates the importance of historical perspectives to enable greater understanding of marine ecosystem dynamics; more accurately gauge contemporary ecosystem health and the effectiveness of restoration measures; and fully appreciate how the legacies of past interactions impact the functioning of marine ecosystems and human communities today. History encourages us to confront our shifting expectations when it comes to the management, conservation and restoration of marine ecosystems. As we contend with swiftly changing oceans and growing human needs in an Anthropocene future, we may never be able to replicate historical ecosystem states, but we can be better informed where to aim, to maximise the benefits for marine ecosystems and ourselves.
